# Heart rate synchrony as a marker of real-world social engagement

**DOI:** 10.1093/pnasnexus/pgag181

**Published:** 2026-06-23

**Authors:** Hanlu He, Jeppe H Christensen, A Josefine Munch Sørensen, Ivana Konvalinka

**Affiliations:** Section for Cognitive Systems, Department of Applied Mathematics and Computer Science, Technical University of Denmark, Kongens Lynby 2800, Denmark; Eriksholm Research Centre, Oticon A/S, Snekkersten 3070, Denmark; Eriksholm Research Centre, Oticon A/S, Snekkersten 3070, Denmark; Section for Cognitive Systems, Department of Applied Mathematics and Computer Science, Technical University of Denmark, Kongens Lynby 2800, Denmark

**Keywords:** interpersonal physiological synchrony, social interaction, sound environment, real-world engagement

## Abstract

Human social behavior unfolds in complex real-world environments influenced by social and environmental factors, yet reliable markers of social engagement and connection remain elusive. Interpersonal physiological synchrony has been proposed as one such marker, but its occurrence in everyday settings is not well established. To investigate the social and environmental factors that influence physiological synchrony, we continuously measured heart rate (HR), GPS, and acoustic features of the sound environment from 72 participants across three multiday trips to New York City, capturing naturalistic social behavior. Across all three trips, HRs reliably synchronized when participants were in close physical proximity, indicating that shared environmental context was sufficient to elicit synchrony. Synchrony was higher among socially familiar peers, and context dependent, emerging during close-proximity interactions and joint attention to shared stimuli, but not dispersed interactions. It was also modulated by the sound environment. Periods with low-to-moderate sound pressure levels and moderate-to-high signal-to-noise ratios were associated with increased synchrony, while periods with excessive environmental noise were related to reduced levels comparable to noninteractive settings, which may reflect lower levels of joint engagement in noisier environments. These findings demonstrate that interpersonal physiological synchrony emerges in naturalistic social settings and is modulated by physical proximity, social familiarity, social context, and the sound environment, establishing it as a reliable marker of real-world social engagement.

Significance Statement Social engagement and connection are fundamental to human well-being, yet difficult to measure in everyday life, particularly in complex real-world environments that influence social interaction and communication. This work shows that interpersonal physiological synchrony—a proposed marker of social engagement—emerges during naturalistic social interactions. Using over 1,000 h of continuous multimodal recordings collected during multiday social experiences in urban settings, we demonstrate that heart rate synchrony varies systematically with physical proximity, social familiarity, shared activities, and the sound environment. By establishing physiological synchrony as a reliable marker outside the laboratory, this study provides a scalable framework for studying social engagement and connection in real-world environments, with implications for understanding social participation and communication in everyday life.

## Introduction

Human social behavior unfolds in complex, dynamic real-world environments, where individuals continuously coordinate their actions, perceptions, and internal physiological signals during shared experiences. One well-documented form of such coordination is interpersonal synchrony, which spontaneously emerges between people’s movements and physiological signals when they interact with one another (1–4), when they passively perceive each other (5) or when they merely have the same sensory input (6–8). For example, people unconsciously synchronize their strides when walking side-by-side (9), and audiences spontaneously synchronize their clapping following a theater or opera performance (10). Similarly, interpersonal physiological synchrony—simultaneous changes in heart rates (HRs) or phase-coupling between people’s periodic cardiac rhythms—accompanies behavioral coordination during face-to-face interactions between mothers and infants and among romantic or marital partners (9, 11, 12), as well as during shared coordinated experiences such as choir singing (13) or playful joint actions (14, 15). Interpersonal physiological synchrony can emerge even without overt behavioral coordination, as shown by the synchronization of heart rhythms between performers and socially affiliated spectators during collective rituals such as fire-walking (16).

Despite widespread evidence for interpersonal synchrony across bodily signals and social contexts, the mechanisms underlying interpersonal physiological synchrony and its potential functional role remain elusive, particularly within the complex, multisensory environments in which everyday social interactions occur. Prior research has consistently linked behavioral synchrony to social bonding and prosocial behavior, showing that people like each other more, feel greater affiliation, and act more cooperatively after synchronizing their movements (17–21). Similarly, physiological synchrony has been shown to be enhanced by social affiliation (22, 23). For example, during a fire-walking ritual, heart-rate coordination was observed only between spectators and performers with preexisting social ties, but not between unrelated dyads (16). Likewise, socially close pairs exhibited stronger heart-rate synchrony than less close or unaffiliated pairs during a high-intensity haunted house experience (23), and autonomic synchrony between individuals meeting on blind dates has been shown to predict subsequent mutual attraction (24).

Synchrony can also arise without direct social interaction or shared social context, such as when people independently watch the same films or listen to the same narratives (25, 26). In such cases, neural and physiological alignment is driven primarily by shared sensory input, and is amplified by simultaneous attentional engagement. For instance, distracted participants exposed to narrative stimuli exhibit reduced intersubject correlation of heart rates (heart rate ISC) (26). Co-presence, such as when individuals watch the same movies together, further amplifies physiological synchrony (5). This is consistent with accounts proposing that physiological and neural synchrony may be markers of joint attention or collective engagement (27–31), which are enhanced by shared social context (32, 33).

However, whether and how physiological synchrony emerges during everyday social interactions outside of controlled laboratory environments remains unclear. Measuring engagement in naturalistic settings is particularly important yet challenging, as subtle disengagement is difficult to detect and may indicate sensory difficulty, especially in individuals with hearing loss or degraded sensory input who experience increased cognitive demands in complex sound environments (34–36). In the absence of overt behavioral synchrony, it is further unclear whether physiological synchrony emerges in low-arousal real-world settings or is instead driven by simultaneous surges in HRs during high arousal events. Establishing whether physiological synchrony emerges during spontaneous face-to-face social exchanges and natural episodes of joint attention, and whether it varies with social affiliation and acoustic features of the sound environment, is therefore essential for evaluating its utility as a marker of real-world social engagement.

To address these questions, we conducted a study in ecologically valid, real-world settings by continuously recording HRs from groups of students engaged in various social activities across three separate 4-day trips to New York (trip 1: *N* = 23, trip 2: *N* = 24, trip 3: *N* = 25), organized as part of Oticon’s annual Audio Explorer events (37). Across the three trips, continuous monitoring generated a large volume of multimodal data, with approximately 293, 367, and 385 total hours recorded per day for trips 1–3, respectively—equivalent to 14–17 h of physiological, spatial, and acoustic data per participant per day. We investigated effects of physical proximity, activity type, social affiliation, and environmental noise on physiological synchrony. We categorized interactions into three distinct activity types: events involving shared sensory stimuli (eg attending the same lecture), face-to-face interactions (eg group games), and dispersed group interactions where the participants were together but not necessarily engaged in close interactions with all members of the group (eg having dinner across numerous small tables). In addition, we tracked participants’ real-time GPS coordinates to quantify physical proximity, in order to investigate whether physiological synchrony emerges more broadly when people are together, as a result of the shared environment.

We further examined whether synchrony is modulated by social affiliation, by comparing HR synchrony between dyads with preexisting social ties vs. those without, while controlling for physical proximity. Given the cognitive demands of processing auditory stimuli in noisy environments—which are often associated with heightened cognitive load and reduced listening engagement (38–40)—we examined how such acoustic challenges encountered during various activities in New York City impacted synchrony. Listening engagement may diminish when the perceived value of communication does not outweigh the effort required to understand the other person. To capture the acoustic context, we employed Oticon’s hearing aids attached to participants’ clothing, enabling real-time recording of environmental acoustic features. We then investigated whether elevated sound pressure levels (SPL) and lower signal-to-noise ratios (SNR) in the sound environment, which may contribute to disengagement, attenuate levels of physiological synchrony.

Specifically, we hypothesized that significant levels of interpersonal physiological synchrony, operationalized using measures of heart rate ISC (26), emerge when people are in close physical proximity, and are modulated by the type of activity people engage in. We expected heart rate ISC to be higher during face-to-face interactions involving reciprocal exchange of social signals, as well as during joint attention episodes to shared sensory stimuli, compared to dispersed interactions or physical separation. In addition, we hypothesized that social affiliation amplifies heart rate ISC through heightened shared engagement, resulting in higher synchrony between socially affiliated individuals. Finally, we hypothesized that heart rate ISC would be higher in auditory environments characterized by higher SNR and lower SPL, facilitating listening and social engagement. Thus, taking into account the physical and social proximity, the type of social activity and hence amount of shared sensory input, and the sound environment, we aimed to establish physiological synchrony more broadly as a socially and acoustically modulated marker of engagement in real-world environments.

## Results

**Figure 1:**
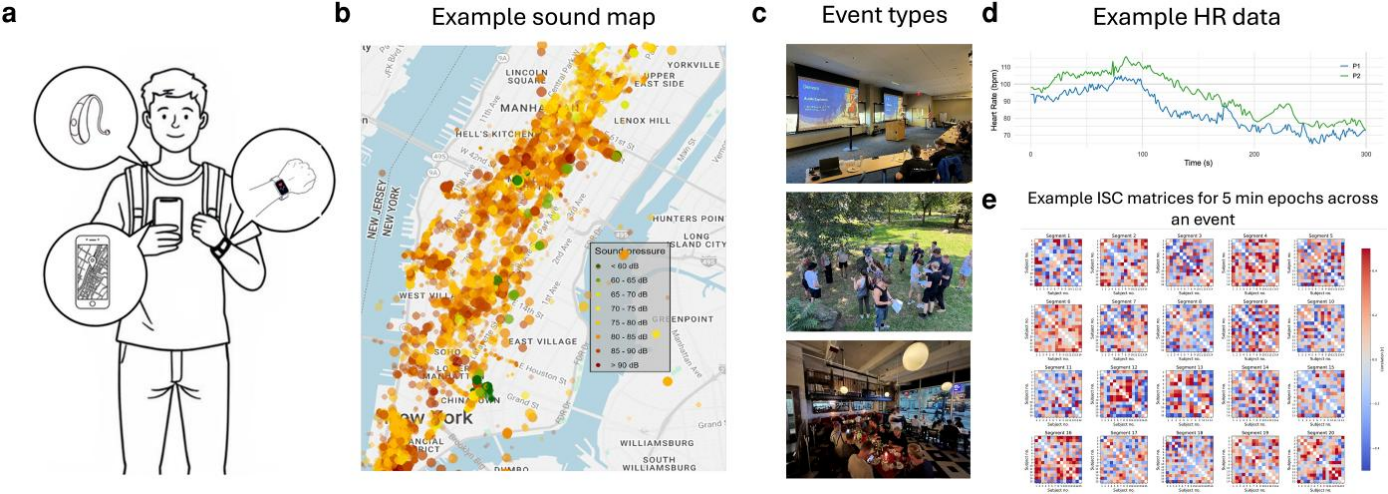
Study design and multimodal data collection during real-world interactions across three trips to New York City. a) Participants wore wearable sensors throughout 4-day trips to New York City: Garmin wristbands to measure HR, hearing aid microphones clipped to the collar to estimate the sound environment, and mobile phones to record GPS coordinates and synchronize sensor streams. b) Example of a “sound-stress” map generated from hearing aid microphones, with colored dots indicating SPL across locations in the city. c) Example photos of the three main event types: a lecture with shared sensory input (stimulus-locked), outdoor group work in Central Park (close proximity face-to-face interaction), and a restaurant setting (dispersed social interaction). d) Example of continuous HR time series from two participants over a 5-min window. e) Heart rate ISC was computed by correlating HR time series across participants in 5-min epochs, illustrated here as correlation matrices across an event. Across three separate trips (n=23,24, and 25 participants), data were collected continuously from 9:00 to 22:00 each day. This enabled us to examine how physiological synchrony varied as a function of shared sensory environments, face-to-face proximity, and more dispersed interactions, while controlling for the sound environment and physical proximity of participants.

Across three separate trips to New York, groups of 23, 24, and 25 student participants, respectively, took part in scheduled activities and had free time to explore the city. Throughout each trip, participants wore hearing aids with microphones and Garmin wristbands synchronized to their mobile phones, which continuously measured features of the sound environment and HRs at various locations (Fig. 1). Data were collected continuously across 4 days for each trip. First, we examined whether heart rate ISC, a proxy for interpersonal physiological synchrony, was modulated by physical proximity between participants. GPS data were used to objectively determine the physical distance between individuals, and to assess heart rate ISC when people were together in groups and in pairs. We further explored whether heart rate ISC was also modulated by social proximity, evaluated as pairs who knew each other beforehand and came to the trip in groups, while controlling for physical proximity. Finally, we investigated heart rate ISC across different planned activities to see whether synchrony was modulated by the amount of shared social or environmental signals, such as settings with shared sensory information, close proximity face-to-face interactions, and dispersed interactions.

### People’s HRs synchronize when they are together in close physical proximity

**Figure 2:**
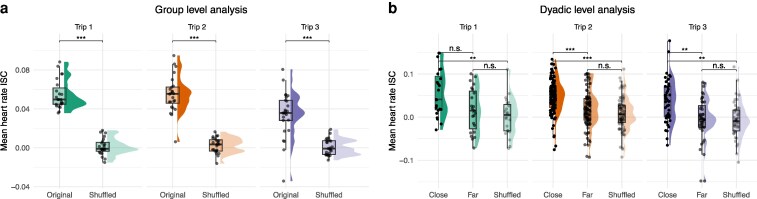
Heart rate synchrony emerges when people are in close physical proximity. a) Group-level heart rate ISC across all 5-min segments when participants were physically together, defined using GPS proximity data during daily activities (9:00–22:00). Average heart rate ISC values were significantly greater than those obtained from time-misaligned shuffled controls (see Quantifying interpersonal HR synchrony section) across all three trips. b) Pairwise comparisons under three physical proximity conditions: close (<20 m), far apart (>1 km), and time-misaligned shuffled control. Across all three trips, heart rate ISC was significantly higher when individuals were in close proximity compared to time-misaligned shuffled controls, with moderate to large effect sizes. For trips 2 and 3, dyadic-level synchrony was also significantly higher when in close proximity compared to far proximity. No differences were observed between far and time-misaligned shuffled conditions across all trips. These findings show that interpersonal physiological synchrony emerges between people in close physical proximity, both at the group and dyadic level. Significant differences are denoted with asterisks (*:P<0.05, **:P<0.01, ***:P<0.001).

To examine whether synchrony arises from mere physical co-presence, independent of social contexts (5), we used GPS data to identify periods when participants were physically close (within 20 m of each other) during daily activities (9:00–22:00) and segmented them into 5-min windows. Across all three trips, heart rate ISC was significantly higher during close-proximity group periods compared to time-misaligned shuffled controls—which reflected synchrony between time-misaligned signals—with large effect sizes (trip 1: t(22)=14.36, $p=1.18\times 10^{-12}$, d=2.94; trip 2: t(23)=9.29, $p=2.98\times 10^{-9}$, d=1.9; trip 3: t(24)=6.76, $p=5.41\times 10^{-7}$, d=1.35) as shown in Fig. 2b.

We then examined physiological synchrony at the dyadic level to better control for the physical proximity. Each pair was compared across three conditions: close proximity (within 20 m), far apart (more than 1 km), and time-misaligned shuffled controls. Repeated-measures ANOVAs revealed a consistent main effect of proximity across all trips (trip 1: F(2,44)=7.21, p=0.002, partial $\eta^{2}=0.18$; trip 2, F(2,184)=20.48, $p=9.29\times 10^{-9}$, partial $\eta^{2}=0.13$; trip 3, F(2,88)=8.92, p=0.001, partial $\eta^{2}=0.12$.). Post hoc tests confirmed that heart rate ISC was reliably higher when pairs were in close proximity compared to time-misaligned shuffled controls (trip 1: t=4.03, $p_{\mathrm{Bonf}.}=0.002$, d=1.08; trip 2: t=6.26, $p_{\mathrm{Bonf}.}=3.54\times 10^{-8}$, d=1; trip 3: t=3.51, $p_{\mathrm{Bonf}.}=0.003$, d=0.74).

For comparisons between close and far conditions, trips 2 and 3 showed significant differences (trip 2: t=4.24, $p_{\mathrm{Bonf}.}=1.61\times 10^{-4}$, d=0.57; trip 3: t=3.4, $p_{\mathrm{Bonf}.}=0.004$, d=0.79), while for trip 1, the difference was not significant (t=2.32, $p_{\mathrm{Bonf}.}=0.09$, d=0.76). Further, no significant differences were observed between the far and time-misaligned shuffled conditions (trip 1: t=−1.18, $p_{\mathrm{Bonf}.}=0.75$, d=−0.34; trip 2: t=−2.3, $p_{\mathrm{Bonf}.}=0.07$, d=−0.35; trip 3: t=1.55, $p_{\mathrm{Bonf}.}=0.38$, d=0.3), as seen in Fig. 2b.

Together, these results demonstrate that HR synchrony emerges when individuals are in close physical proximity, both at the group and dyadic level, suggesting that shared environmental input facilitates physiological synchrony in real-world environments.

### Physiological synchrony is modulated by social familiarity

The effect of social familiarity—defined as whether participants knew each other prior to the trip—was examined to test whether physiological synchrony was modulated by preexisting relationships beyond physical co-presence. Pairs were categorized as familiar (participants who knew and worked with each other before the trip) or unfamiliar (participants who were strangers prior to the trip). To control for physical proximity, pairwise heart rate ISC values were computed only for segments when both participants were physically together identified by GPS coordinates in the same group and then averaged across those group segments.

**Figure 3:**
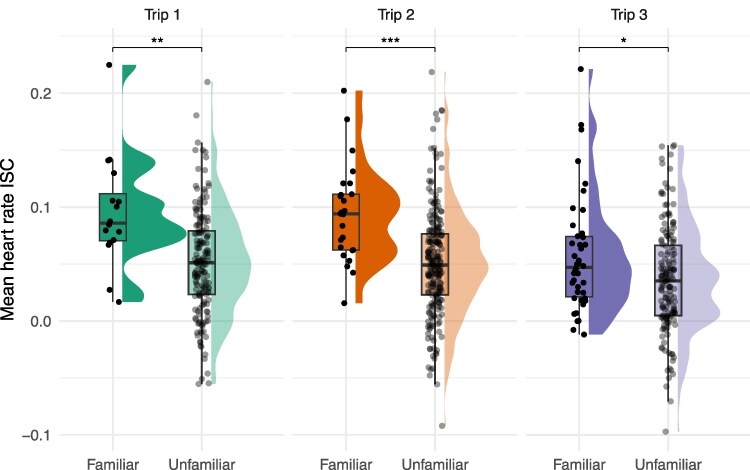
Comparison of heart rate ISC between familiar and unfamiliar pairs across three trips. Mean heart rate ISC values are shown for familiar pairs (participants who knew each other prior to the trip) and unfamiliar pairs (participants with no prior relationship), averaged across all group settings. Across all three trips, familiar pairs exhibited significantly higher heart rate ISC than unfamiliar pairs, indicating that social proximity—defined by preexisting social familiarity—enhances physiological synchrony beyond the effect of physical co-presence. Significant differences are denoted with asterisks (*:P<0.05, **:P<0.01, ***:P<0.001).

The results revealed significantly higher heart rate ISC for familiar pairs compared to unfamiliar pairs across all three trips (trip 1: W=3.45, p=0.0029, rank-biserial r=0.95, trip 2: W=4.68, $p=2.84\times 10^{-6}$, r=0.32, trip 3: W=2.38, p=0.018, r=0.17), as seen in Figure 3. These findings indicate that social familiarity enhances HR synchrony even when controlling for physical proximity, suggesting that preexisting relationships facilitate physiological alignment.

### Physiological synchrony across different social contexts

**Figure 4:**
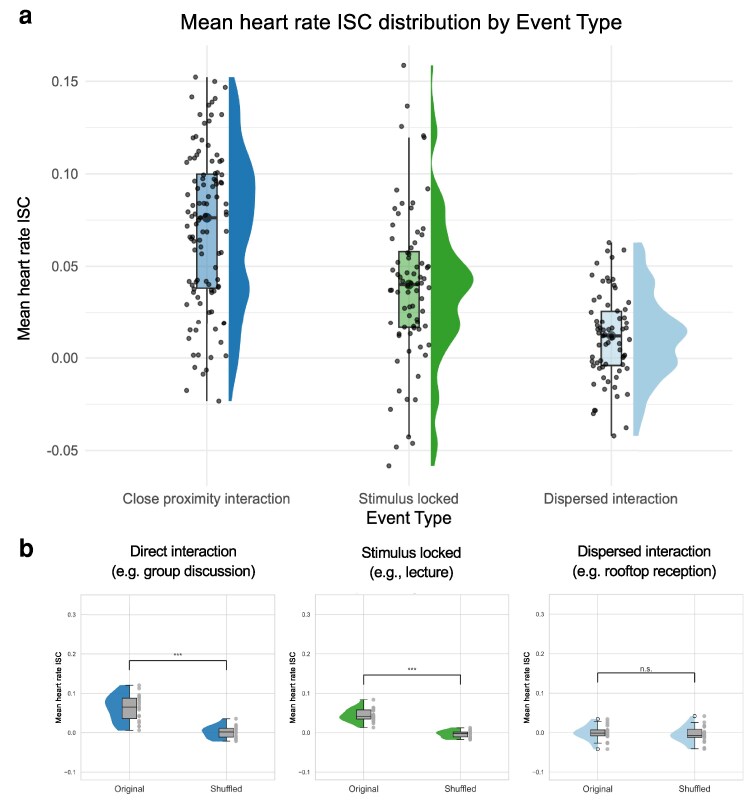
Physiological synchrony across different social interaction contexts. a) Distribution of mean heart rate ISC across participants during three types of interaction: close proximity interaction (eg group discussion or games), stimulus-locked interaction (eg attending a performance or lecture), and dispersed interaction (eg rooftop reception). Heart rate ISC was highest during close proximity interactions, intermediate during stimulus-locked interactions, and lowest during dispersed interactions. b) Example events corresponding to the three categories. Each dot represents the mean heart rate ISC of one participant with all others across 5-min segments within the event, compared against a time-misaligned shuffled control generated by random pairing of time segments. Significant levels of synchrony were observed during close proximity and stimulus-locked events, but not during dispersed interactions. Asterisks denote significant differences (Wilcoxon signed-rank test with Bonferroni correction; *:P<0.05, **:P<0.01, ***:P<0.001).

To examine how different types of interactions relate to interpersonal physiological synchrony, events were first manually labeled based on their social and contextual characteristics. Because activities varied widely in structure and social engagement, they were grouped into three categories reflecting shared behavioral and attentional contexts: close-proximity interactions (eg group discussions during games or meals), where participants actively engaged in face-to-face interactions; stimulus-locked interactions (eg attending lecture presentations or shows), where everyone jointly attended to an external sensory input; and dispersed interactions (eg receptions or co-located activities without shared engagement), where participants were more scattered and interactions were brief (Fig. 4).

Across all three trips, significant group-level heart rate ISC was observed primarily during close-proximity and stimulus-locked events, but not during dispersed interactions. For example, in trip 1, heart rate ISC was significantly elevated compared to time-misaligned control during *Social Dinner 2*, where participants sat close together and engaged in conversation (t=4.8, $p=2.9\times 10^{-4}$, d=1.24), and during *Presentation 1*, where participants jointly attended a science lecture (W=9.2, $p=1.26\times 10^{-8}$, d=2). In contrast, no significant differences were observed for *Casual Reception 1*, where participants were scattered around a rooftop terrace (t=1.09, p=0.31, d=0.36).

A similar pattern was found in trip 2 and trip 3, with presentation-based and interactive events in close proximity consistently eliciting high heart rate ISC. For instance, in trip 3, *Presentation 7* (joint attention to a shared stimulus) showed very high levels of synchrony (t=11, $p=1.02\times 10^{-8}$, d=2.33), as did *Group Game 2*, where participants engaged in intense group discussions to solve an engineering task (t=5.51, $p=1.7\times 10^{-4}$, d=1.26). In contrast, dispersed settings such as *Casual Dining 1–4* and *Casual Reception 1–2*, where participants were seated apart and loosely engaged, exhibited low and nonsignificant heart rate ISC levels.

Importantly, these effects were not explained by differences in mean heart rate. While no global correlation was found between mean HR and heart rate ISC (r=0.04,p=0.45), a more detailed analysis of specific interaction types showed that mean heart rate was not associated with heart rate ISC in either close-proximity (r=0.08,p=0.30) or dispersed (r=0.15,p=0.09) settings. However, within the stimulus-locked condition, when excluding the two extreme outliers, we saw a significant positive relationship (r=0.36,p<0.001). This could suggest that in contexts of shared attention, higher arousal or engagement may lead to increased physiological alignment. Furthermore, as shown in Fig. S2, both close-proximity and dispersed interactions resulted in similarly elevated mean HR, yet only the former exhibited significant levels of synchrony, while stimulus-locked events exhibited the highest levels of heart rate ISC despite having the lowest overall mean HR.

Additionally, we assessed whether these results could be attributed to systematic differences in signal integrity across event types. We quantified data quality as the percentage of long gaps (intervals >5 s) in the raw HR signal for each participant. Statistical analysis revealed no significant correlation between the amount of missing data and heart rate ISC ($\beta =3.22\times 10^{-4}$, p=0.79), nor did missing data levels differ significantly across the three event types (p=0.32). These results indicate that there is no systematic difference in the findings explained by data quality. Full statistical details for all events and data quality analyses are provided in the Supplementary information (Notes S7–S9; Figs. S1–S6; Tables S2–S4).

### The effect of sound environment on physiological synchrony

**Figure 5:**
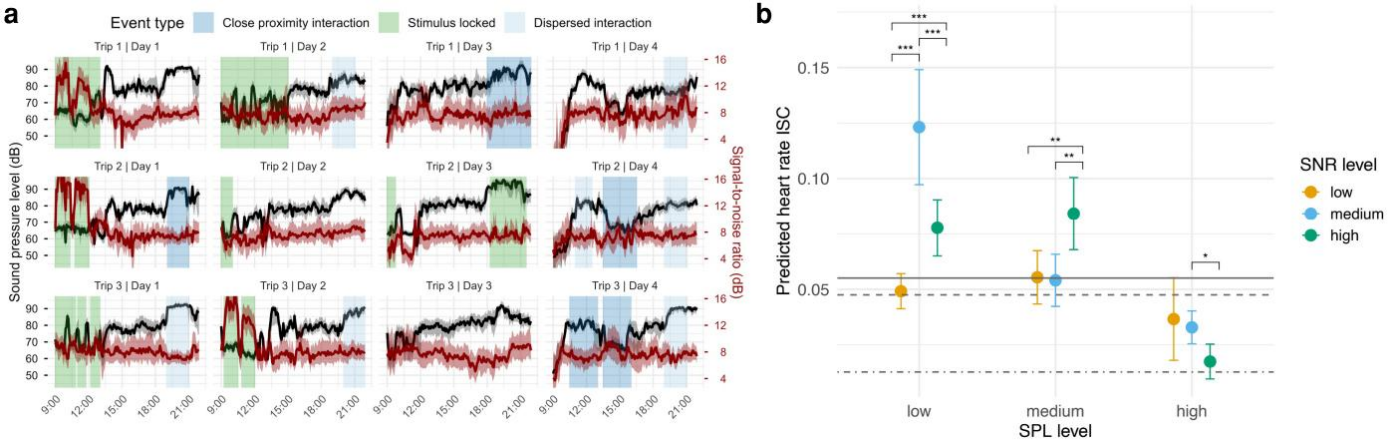
Sound environments and their relationship to physiological synchrony. a) Median SPL (left *y*-axis) and SNR (right *y*-axis) in decibels for each trip (rows) and day (columns) across all participants estimated in 5-min windows. The shaded area around each value represents the interquartile range. Vertical shaded areas represent periods of different social interaction contexts, color-coded by Event Type. b) Predicted heart rate ISC values across combinations of SPL (low, medium, high) and SNR (low, medium, high) estimated from a linear model including SPL, SNR, their interaction, and mean HR as a covariate. Points and error bars show estimated marginal means ±95% CI for each SPL × SNR cell. Gray horizontal lines indicate mean heart rate ISC observed in different event contexts (solid = close-proximity interaction, dashed = stimulus-locked, dot-dash = dispersed). Synchrony varied as a function of SPL and SNR: in quiet environments (low SPL), synchrony peaked at medium SNR ; at moderate SPL, synchrony was strongest at high SNR, with no difference between low and medium SNR; in loud environments (high SPL), synchrony was generally lower, comparable to levels observed during dispersed interactions.

We examined whether exposure to different sound environments, characterized by SPL and SNR, was associated with variations in physiological synchrony. To contextualize the variability in acoustic sensory experience across the study, we visualized SPL and SNR over time for each trip. Figure 5a shows moment-to-moment and day-to-day fluctuations in the sound environment participants encountered, with color-coded background segments indicating when different social context event types occurred. It shows that the sound environments were relatively consistent across the three trips.

SPL and SNR values were estimated using participants’ hearing aids and categorized into low, medium, and high levels based on tertiles within participants. Heart rate ISC was modeled using a linear model including SPL level, SNR level, their interaction, with mean HR as a covariate (mean_ISC∼SPL_level×SNR_level+mean_HR). Inclusion of mean HR slightly improved explained variance ($R^{2}$ increased from 0.0265 to 0.0286), but the effect was small, indicating that general arousal contributed minimally to levels of physiological synchrony.

Examination of the model revealed a significant interaction between SPL and SNR, indicating that the relationship between acoustic clarity (ie SNR) and synchrony depends on the overall sound level. The following results describe this interaction in detail (Fig. 5b).

#### Low SPL (quiet environments)

Heart rate ISC peaked at medium SNR, followed by high SNR, and was lowest for low SNR. Pairwise contrasts showed that medium SNR was significantly higher than low SNR ($\hat{\Delta }=-0.0697$, SE=0.0106, t(5,886)=−6.59, P<0.0001) as well as high SNR ($\hat{\Delta }=-0.0410$, SE=0.0109, t(5,886)=−3.77, P=0.0005). Heart rate ISC at high SNR was also significantly higher than low SNR ($\hat{\Delta }=0.0287$, SE=0.0075, t(5,886)=3.83, P=0.0004).

#### Medium SPL (moderately loud environments)

Heart rate ISC at high SNR was significantly higher than at low or medium SNR ($\hat{\Delta }$ (high–low, high–medium) = (0.02875, 0.03086), SE (high–low, high–medium) = (0.00831, 0.00903), t(high–low, high–medium) = (3.416, 3.458), *p*(high–low, high–medium) = (0.0016, 0.0019), while low and medium SNR did not differ ($\hat{\Delta }=0.00211$, SE=0.00832, t(5886)=0.254, P=1.000).

#### High SPL (loud environments)

Heart rate ISC values were generally lower across all SNR levels. Only the contrast between high and medium SNR reached significance ($\hat{\Delta }=-0.01818$, SE=0.00744, t(5,886)=−2.443, P=0.0437); other contrasts were not significant (high–low: P=0.2264; low–medium: P=1.000).

This pattern suggests that low-to-medium SPL combined with sufficient acoustic clarity (medium-to-high SNR) associates with stronger physiological alignment, whereas louder and noisier environments are linked to reduced synchrony. In particular, the noisiest conditions produced heart rate ISC levels comparable to those observed during dispersed interactions, indicating that such environments may coincide with less interactive or socially engaging moments.

## Discussion

In this study, we demonstrate that interpersonal physiological synchrony, as indexed by heart rate ISC, emerges robustly in everyday social interactions and is modulated by both social and environmental factors. Across three multiday trips, HR synchrony was consistently higher when participants were in close physical proximity, when they interacted with familiar peers, and during activities that fostered shared attention and engagement, such as group games and attending lectures. In contrast, synchrony was reduced during dispersed interactions where opportunities for reciprocal social exchange or shared sensory input were more limited. Moreover, features of the sound environment further modulated levels of synchrony: favorable listening conditions (low-to-medium SPL and medium-to-high SNR) were associated with higher synchrony, suggesting that listening conditions might influence the degree of physiological alignment. Importantly, these effects were replicated across the three studies (conducted across different years), and could not be attributed to higher global arousal as we found no association between heart rate ISC and mean HR.

While interpersonal physiological synchrony is a well-documented phenomenon that occurs when individuals engage in both direct and indirect interaction (5, 26, 41, 42), most studies remain confined to lab settings or high-arousal contexts (16, 23). To better understand physiological synchrony in naturalistic contexts, we analyzed HR data collected via wearable sensors during three different multiday real-world trips, capturing both scheduled activities and free-roaming exploration.

Our results demonstrate that people’s HRs synchronize when they are in close physical proximity. GPS-based measures of spatial distance revealed that both group- and dyad-level heart rate ISC were higher when participants were in close physical proximity of each other, compared to time-misaligned control analyses. This pattern was robust across all three trips with different participants, suggesting that shared physical space alone can facilitate physiological coupling in real-world environments. However, the result was less robust across trips for the dyad-level close–far comparison. While dyads in trips 2 and 3 showed higher heart rate ISC when they were close together than when they were far apart, the difference was not significant for trip 1. Notably, the effect sizes for the dyad-level (trip 1: d=0.76; trip 2: d=0.57; trip 3: d=0.79) comparisons were smaller than those observed at the group level (trip 1: d=2.94; trip 2: d=1.9; trip 3: d=1.35). This may be because close proximity at the dyadic level does not necessarily indicate that partners were interacting or processing the same information, whereas proximity at the group level more likely reflected participation in a shared activity. This weaker effect in trip 1 may thus be explained by lower effect size and the substantially smaller amount of usable data. Specifically, the number of exact matching pair segments with sufficiently long continuous data for the close vs. far conditions was much lower in trip 1 (1,262 vs. 1,163) compared with trips 2 (9,542 vs. 8,682) and 3 (2,904 vs. 3,385).

Beyond physical proximity, social familiarity was also found to modulate physiological synchrony. Pairs who knew each other prior to the trip exhibited higher heart rate ISC than unfamiliar pairs, while controlling for physical proximity, indicating that preexisting familiarity enhances alignment. This effect may reflect greater shared engagement to common stimuli or enhanced face-to-face interaction among familiar individuals. Interestingly, physiological synchrony was robust enough to capture this effect, despite all participants (familiar and unfamiliar) being in close proximity, and hence exposed to common environmental stimuli.

Examining physiological synchrony under different social contexts revealed that people’s HRs tend to synchronize during close-proximity interactions (eg group discussions) and when co-attending to common stimuli (eg watching a lecture or performance), but not during dispersed interactions (eg sitting scattered in a restaurant). The observed group heart rate ISC during stimulus-driven events aligns with previous laboratory research showing heart-rate synchrony between people listening to common narrative stimuli (26), and real-world studies on physiological entrainment to music during live concerts (43). Our findings thus indicate the context-dependent nature of physiological synchrony, which could be related to differences in participants’ levels of attentional engagement. For instance, during collaborative group activities, participants may have felt more motivated to engage in frequent, dynamic verbal exchanges compared to less interactive settings. This heightened reciprocity in interpersonal communication might have contributed to the observed higher degree of alignment in physiological responses.

The data also show that heart rate ISC was not associated with mean heart rate in either close-proximity or dispersed settings. Furthermore, while close-proximity and dispersed interactions resulted in similarly elevated mean HRs, only the former exhibited significant synchrony, while stimulus-locked events exhibited the highest levels of synchrony despite having the lowest overall HR. Within stimulus-locked contexts, we did observe a positive relationship between mean HR and synchrony after excluding outliers. This finding aligns with established cases of physiological and neural synchrony observed during shared attentive states in educational (30), dance watching (44), and music listening contexts (45). These results suggest that in environments involving joint attention to an external source, higher levels of engagement or arousal may correspond to increased physiological alignment.

Further, we examined how variations of acoustic features in the sound environment were associated with the strength of physiological synchrony across all activities. The significant interaction between SPL and SNR indicated that the association between SNR (eg the clarity of the sound environment) and physiological synchrony depended on the overall loudness of the environment. In quieter settings (low SPL), heart rate ISC was highest at medium SNR, while at moderate SPL heart rate ISC was highest for high SNR. In louder environments (high SPL), heart rate ISC was generally lower across all SNR levels, with values comparable to those observed during dispersed interactions. We propose three plausible interpretations for the observed impact of the sound environment. One explanation is that environments characterized by loud sound levels (high SPL) and background noise (low SNR) act as a sustained stressor that induces physiological arousal and stress (46, 47). Following the Neurovisceral Integration Model, sustained arousal from noise may reduce the HR’s dynamic range for further fluctuations in response to emotional cues during active listening or communication (48). However, this explanation cannot fully account for the effects as this would require a global negative correlation between mean HR and heart rate ISC, which was not observed. An alternative explanation is that low SNR environments increase the demand for auditory perception and compensation strategies, potentially reducing cognitive resources for interpersonal dynamics (34–36, 40), resulting in reduced physiological synchrony (49). Finally, the relationship may be driven by the nature of the activities themselves. Social activities that lead to high physiological synchrony—such as clear verbal communication—typically occur in environments characterized by high SNR. In this view, high SNR values (and low to medium SPL values) coincide with the social contexts most likely to foster physiological synchrony, rather than directly inducing it.

Importantly, because our data are observational, we cannot determine whether these associations reflect a direct effect of the sound environment on physiological synchrony or whether they are driven by the types of activities that typically occur under different acoustic conditions. One limitation is that we did not measure perceived cognitive effort or listening engagement directly, for example via experience sampling. Thus, while our findings show that SPL and SNR are associated with variations in interpersonal physiological synchrony, the underlying mechanisms remain speculative.

While the study’s ecological validity is a key strength, it also presents with some inherent limitations. First, there is an inevitable trade-off in data quality when conducting research in real-world settings, as heart rate data were collected using wristbands instead of the raw cardiac activity data typically obtained from electrocardiograms in laboratory environments. The wristbands are less accurate and more susceptible to motion artifacts and variations in activity types. Additionally, optimal GPS tracking using mobile phones requires an unobstructed view of the sky (50), whereas in our case, the participants spent considerable time indoors. These limitations in data quality necessitated extensive preprocessing decisions (cleaning, resampling, and interpolation). Additionally, we were limited by the technical specifications of the acoustic data collection. The hearing aid sound data were sampled every 20 s, which precluded high-resolution voice activity detection or speech decomposition analysis. While high SNR environments often identify the acoustic clarity necessary for verbal communication, the SNR estimators are optimized for low power consumption and real-time processing rather than perfect speech isolation. Thus, we cannot definitively compute the relative amounts of mixed speech for different activities. The scarcity of prior literature addressing these methodological challenges made it particularly difficult to establish standardized methods for handling such data in real-world contexts.

There was also limited control over how and when the participants interacted with each other aside from having the timestamps of planned activities. Consequently, we could not precisely identify the type of interaction each participant was engaged in when in close physical proximity to others. This constraint made it challenging to distinguish between reciprocal interaction and stimulus locking as drivers of interpersonal physiological synchrony, leaving us to make inferences and generalize based on event labels.

Furthermore, we assessed whether systematic differences in signal integrity across activities could explain our findings. We quantified data quality as the percentage of “long gaps” (intervals >5 s) in the raw HR signal for each participant. Statistical analysis revealed no significant correlation between the amount of missing data and heart rate ISC, nor did missing data levels differ significantly across interaction categories. These results indicate that there is no systematic difference in the findings explained by data quality. However, it remains possible that other factors inherent to naturalistic social contexts, such as synchronized body movements or the temporal structure of verbal communication, contributed to the observed physiological alignment. While our current data do not allow for a full decomposition of these behavioral components, the lack of association between signal dropout and synchrony suggests that the findings are not simply an artifact of varied data quality across different social settings.

Since our study investigates HR synchrony in naturalistic interactions, it is not designed to disentangle exact mechanisms that underlie physiological synchrony. While entrainment of neural rhythms to common stimuli has been well documented and extensively studied, physiological rhythms do not serve the same perceptual functions. Rather than a direct perceptual response, we speculate that HR synchrony in naturalistic settings represents an alignment in autonomic states that arises when individuals share similar levels of attention, arousal, or emotional regulation. While shared sensory input can contribute to this synchrony, it appears to do so primarily when the input is consciously processed and similarly interpreted, such as during narratives or socially shared experiences (5, 8, 26). This shared engagement likely relates to heart rate ISC in two ways. First, during stimulus-locked events, participants’ autonomic nervous systems may be simultaneously modulated by the temporal structure and emotional content of external input. This process of simultaneous regulation and arousal in response to emotional events may be further amplified by social presence; for instance, HR synchrony is higher when people co-attend to a stimulus in a shared context than when they are alone (5). This is consistent with our finding that within joint stimulus-locked settings, higher arousal (mean HR) was associated with increased physiological alignment. Second, in close-proximity interactions, synchrony may emerge through the reciprocal exchange of social signals—such as speech prosody, facial expressions, and gestures—which involve mutual physiological regulation. This aligns with findings from romantic blind date settings showing that physiological synchrony captures a “genuine emotional exchange” through subconscious alignment more effectively than visible behaviors like smiling or mimicry (24). Furthermore, as seen in studies of strangers tasked with cooperating or competing, the meaning of this synchrony is tied to the social context and how much individuals’ bodies are actually reacting to the situation (51). This could explain nonsignificant HR synchrony in dispersed settings in our study, even when the overall arousal level (indexed by mean HR) was comparable to close-proximity interactions (Fig. S2). Hence, heart rate ISC appears to be also driven by shared cognitive and emotional regulation, which is often heightened in social settings, rather than low-level stimulus locking alone. That said, future research is required to further isolate these physiological mechanisms from direct perceptual entrainment.

The present findings highlight the potential of interpersonal physiological synchrony as a scalable, ecologically valid index of shared engagement in everyday life. By combining wearable sensing, environmental acoustics, and social context labels with measures of interpersonal synchrony, our approach provides an index of collective behavior in real-world environments. Importantly, our results are robust, replicating across three different trips and studies. This framework has broad applicability for quantifying real-world social dynamics. Measures of physiological synchrony can be used to track moment-to-moment fluctuations in collective engagement, to study how social connection emerges and evolves across contexts such as education and collaboration, and to index listening engagement and participation in individuals with sensory challenges such as hearing loss. More broadly, combining physiological synchrony with environmental and behavioral data offers a new approach toward social sensing—characterizing how humans co-regulate their physiological states within complex social and sensory environments.

## Methods

### Participants and ethics

Seventy-two participants (60 male; age range 19–32 years, M=23, SD = 2.09) took part in three 4-day study trips to New York City (23, 24, and 25 participants per trip). Participants were recruited through three editions of the Audio Explorer Challenge (37), a competition hosted by Oticon A/S. They entered the competition in preformed teams consisting of 2 to 5 individuals, with the winning teams rewarded with participation in a study trip to New York City. Participants provided informed consent for the use of anonymized data in aggregated analyses and agreed that data could be stored on Oticon A/S-owned secure servers. Additional consent was obtained for access to GPS location and health data via Apple HealthKit. All procedures followed a privacy-by-design framework in compliance with the General Data Protection Regulation (EU 2016/679). The Danish Capital Region Scientific Ethical Committee determined that this study did not constitute health research under the Act on Research Ethics Review of Health Research Projects and therefore did not require ethical approval (reference number F-25048245).

### Study design and data collection

Data were collected during three separate 4-day trips to New York City. Participants engaged in a combination of preplanned group activities and unstructured free time, enabling a wide range of real-world social interactions. Continuous multimodal data were collected between 9:00 AM and 10:00 PM each day. Every 20 s, hearing aids (Oticon Opn S) recorded estimates of the surrounding sound environment, while wristbands (Garmin VivoSmart 4) measured instantaneous HR at each detected heartbeat using pulse plethysmography. Smartphones (iPhone 7) recorded GPS location data. Event timelines were documented by the research team and combined with GPS data to label social context and identify periods group interaction based on physical proximity. Acoustic feature extraction and signal-processing details for the hearing aid microphones are provided in Supplementary information S1. Technical characteristics and limitations of the HR sensors are described in Supplementary information S2.

### Preprocessing

#### GPS data

Location logs with invalid coordinates were removed. Data were restricted to the Greater New York Area using predefined latitude (40.0–45.0) and longitude (−75.0 to −72.0) boundaries. Spatial distances and time differences between consecutive samples were computed using the Haversine formula to derive speed estimates. Movement status was classified as stationary or moving using a 2 m/s threshold, with stationary points retained for analysis. Large spatial jumps (>30 m within 20 s) were identified and interpolated to reduce GPS artifacts.

#### Heart rate data

Instantaneous HR data were resampled to 10 Hz and interpolated using Piecewise Cubic Hermite Interpolating Polynomials, which minimizes error in wearable HR signals (52). Data were segmented into nonoverlapping 5-min epochs corresponding to events, group periods, and dyadic interaction periods.

The 5-min window length was selected based on systematic comparisons with 3- and 10-min windows across all datasets. Shorter windows were unsuitable due to the low sampling rate of physiological and acoustic measures, whereas longer windows reduced sensitivity to short-term interpersonal dynamics. The 5-min window provided the best balance between temporal resolution, data quality, and participant inclusion, yielding consistent data retention across trips (91.8, 87.7, and 92.4%).

### Analysis

#### Physical proximity

Physical proximity was assessed at both group and dyadic levels using GPS data. Group periods were defined as intervals of at least 30 min during which the average interpersonal distance among participants was below 100 m. This threshold was empirically motivated: during known close-proximity events (eg meals, presentations), mean group distances ranged from 0.08 to 0.15 km, whereas free-time periods showed substantially larger and more variable distances (0.97–2.45 km). The 100 m criterion thus reliably distinguished structured group activities from dispersed periods while accommodating spatial spread within shared environments.

At the dyadic level, participant pairs were classified as close when separated by <20 m and far when separated by more than 1 km for periods lasting at least 15 min. The 20 m threshold reflects observed proximity during seated or constrained interactions and accounts for the typical horizontal accuracy of smartphone GPS in urban settings (7–13 m) (50).

#### Social familiarity

Social familiarity was defined based on preexisting team membership. Participants who entered the study as part of the same team were classified as familiar, whereas participants from different teams were classified as unfamiliar. Familiarity analyses were restricted to periods of close physical proximity. Specifically, we categorized interactions within the group as familiar interactions and those involving members of different teams as unfamiliar interactions. This distinction allowed us to explore the relationship between physical and social proximity and assess how team membership influenced patterns of interaction during group settings.

### Quantifying interpersonal HR synchrony

Interpersonal physiological synchrony was quantified using heart rate ISC, a measure used to assess temporal alignment of physiological signals across individuals exposed to shared contexts or stimuli (26, 27). Heart rate ISC captures similarity in slow HR fluctuations over time and provides a simple, interpretable measure that is robust to noise and well suited for long-duration, naturalistic recordings. A detailed description of signal segmentation, normalization, and heart rate ISC computation at the group, dyadic, and familiarity levels is provided in Supplementary information S3.

Heart rate time series were segmented into nonoverlapping 5-min windows and z-scored within each segment to remove interindividual differences in mean HR and variance. Heart rate ISC was computed as the Pearson correlation between time-aligned HR signals from participant pairs, yielding one synchrony value for each segment.

We computed synchrony at multiple analytical levels. At the group level, each participant’s HR segment was correlated with those of all other participants present in the same group period, and correlations were averaged to yield a single heart rate ISC value per participant. At the dyadic level, heart rate ISC was computed for participant pairs during periods of close proximity and during periods when pairs were spatially separated. For social familiarity analyses, heart rate ISC was computed separately for familiar and unfamiliar pairs during periods of close proximity.

To assess whether observed heart rate ISC exceeded chance levels, we implemented a time-misaligned shuffled control analysis in which heart rate segments were randomly paired across participants such that paired segments did not overlap in time. The number of random pairings matched that of the observed time-aligned data. Heart rate ISC from time-misaligned pairs provided a control measure of synchrony expected in the absence of shared temporal structure. The full procedure for time-misaligned control analysis is described in Supplementary information S4.

#### Modeling the effects of sound environment on physiological synchrony

To assess whether the sound environment modulated HR synchrony, data across all three trips were pooled. For each group period, median SPL and SNR were computed across participants for each 5-min segment (53). SPL and SNR were categorized into tertiles (low, medium, high) to summarize the acoustic context experienced during group interactions.

Group-level heart rate ISC was modeled using linear regression, predicting mean ISC per segment from SPL level, SNR level, and their interaction. In an additional model, mean HR was included as a covariate to account for general arousal. Model comparisons were performed using ANOVA, Akaike information criterion, and changes in $R^{2}$. Full model specifications, categorization thresholds, and statistical results are reported in Supplementary information (S5, Table S1).

### Statistical analysis

Statistical analyses tested whether heart rate ISC exceeded time-misaligned control levels and whether synchrony varied as a function of physical proximity, social familiarity, social context, and sound environment. Assumptions of normality were assessed prior to testing, and appropriate parametric or nonparametric tests were applied. Effect sizes were reported throughout. Additional details regarding statistical testing, model comparisons, and evaluation metrics are provided in Supplementary information S6.

## Supplementary Material

pgag181_Supplementary_Data

## Data Availability

The dataset underlying this article is available at https://osf.io/cswt2/overview?view_only=364e00ef2c4e4b17a34334d43cf5a3d8. The code for replicating the analyses underlying this article is available at https://osf.io/cswt2/overview?view_only=364e00ef2c4e4b17a34334d43cf5a3d8.

## Preprints

A preprint of this article is published at https://doi.org/10.64898/2025.12.01.691366.
