# Supplementary material for: Heart rate synchrony as a marker of real-world social engagement
Source: PNAS Nexus. 2026 Jun 23;5(6):pgag181. doi: 10.1093/pnasnexus/pgag181 (PMC13288439; doi:10.1093/pnasnexus/pgag181)
Supplement: pgag181_Supplementary_Data [file pgag181_supplementary_data.pdf]

Supplementary Information for

[Heart rate synchrony as a marker of real-world social engagement]

[Hanlu He, Jeppe H Christensen, A. Josefine Munch Sørensen, Ivana Konvalinka]

Correspondence to: [Hanlu He]
Email: [hahea@dtu.dk].

This PDF file includes:

- Supplementary Notes S1–S9
- Supplementary Figures S1–S6
- Supplementary Tables S1–S4
- Supplementary references

S1. Acoustic data acquisition and feature extraction. The hearing aids logged acoustic data, including SPLs and noise floor (NF) estimates. Unlike laboratory settings, where SNR calculations involve well-defined target signals and noise, SNR in real-world settings relies on estimates from the processed acoustic features sensed by hearing aid microphones. These include environmental sounds, mixed speech, and background noise sources (1, 2).

In this study, the hearing aids used low-pass infinite impulse-response filters with a time constant of 63 milliseconds to estimate SPL and NF. SNR was determined as the difference between the A-weighted (adjusted to mimic human perception of sound as we are not equally sensitive to all frequencies) for SPL and the NF estimate. The SPL represents the immediate sound environment, while the NF represents slower-varying background noise, derived using a 1-second attack and 0.5-second release time for dynamic changes (3). Hearing aid microphones were clipped onto participants' clothing, around the collar area, for consistent data collection.

S2. Heart rate measurement characteristics. The Garmin wristbands use data from optical sensors (pulse plethysmographs) to estimate heart rates in beats per minute (BPM). These sensors emit green light through the skin, which is reflected by red blood cells, allowing the wristband to detect changes in blood flow due to heartbeats. The BPM value provides an average heart rate over short intervals (approximately a few seconds), making the readings suitable for long-term tracking, but provide less precise recordings than electrocardiograms (ECG).

ECG, often considered the gold standard in clinical studies, records the electrical activity of the heart by measuring voltage changes on the skin surface, capturing details like P-waves, QRS complexes, and T-waves (4). In contrast, the optical heart rate monitoring of the Garmin wristbands smoothed out small heart rate variations. This method is sensitive to motion artifacts (5) and the participant's physical activity, which can lead to inconsistent RR-intervals not necessarily caused by ectopic beats. As a result, heart rate variability (HRV) analyses were not possible with this dataset. For more details, see Garmin's official heart rate monitoring guide (link) and accuracy disclaimer (link).

S3. Detailed quantification of interpersonal heart rate synchrony. Interpersonal physiological synchrony was quantified using inter-subject correlation of heart rate (heart rate ISC), which assesses the similarity of temporal fluctuations in physiological signals across individuals (6, 7). This approach captures shared temporal structure without requiring precise event locking and is robust to noise, making it suitable for long-duration naturalistic recordings.

For each participant, instantaneous heart rate time series were segmented into non-overlapping 5-minute windows. Within each window, signals were z-scored to remove inter-individual differences in mean heart rate and variance. heart rate ISC was computed as the Pearson correlation between time-aligned heart rate segments.

heart rate ISC was computed at multiple analytical levels:

- **Group-level analysis:** Each participant's heart rate segment was correlated with the time-aligned segments of all other participants present during the same group period. Correlations were averaged across segments and partner participants to yield a single heart rate ISC value per participant.
- **Dyadic proximity analysis:** heart rate ISC was computed for all participant pairs during periods of close physical proximity and during periods when pairs were spatially separated. Correlations were averaged across segments to yield one heart rate ISC value per dyad and condition.
- **Social familiarity analysis:** During periods of close proximity, heart rate ISC was computed separately for familiar pairs (participants from the same team) and unfamiliar pairs (participants from different teams).

S4. Time-misaligned shuffled control analysis. To estimate baseline synchrony expected in the absence of shared temporal structure, we implemented a time-misaligned shuffled control analysis. For each analytical level, heart rate segments were randomly paired across participants such that paired segments did not overlap in time. The number of time-misaligned pairings matched the number of time-aligned pairings used in the observed analyses.

For group-level analyses, each participant's heart rate segments were paired with randomly selected, non-overlapping segments from other participants. For dyadic analyses, time-misaligned segments were generated separately for each participant pair, preserving the number of segments per condition. heart rate ISC values computed from these time-misaligned pairings served as a control distribution reflecting synchrony arising from shared signal properties rather than genuine temporal co-fluctuation.

S5. Modeling the effects of the sound environment on heart rate ISC. Associations between the sound environment and interpersonal heart rate synchrony (heart rate ISC) were examined using pooled data from all three study trips. For each group interaction period, median sound pressure level (SPL) and signal-to-noise ratio (SNR) were computed per 5-minute segment by aggregating values across participants (8). These measures were derived from hearing-aid microphones sampling the sound environment every 20 seconds.

To facilitate interpretation and reduce sensitivity to outliers, SPL and SNR were categorized into tertiles reflecting low, medium, and high acoustic conditions. Categorization thresholds were as follows: SPL — low: 38–67.49 dB, medium: 67.66–81.86 dB, high: 81.88–95.54 dB; SNR — low: −2.32–5.65, medium: 5.67–7.17, high: 7.19–17.86.

Group-level heart rate ISC was quantified as the mean ISC across participants within each segment. A series of nested linear models were fit to predict mean heart rate ISC. Model 1 included additive effects of SPL and SNR. Model 2 additionally

included the SPL × SNR interaction. Model 3 further included mean heart rate (HR) as a covariate to account for general physiological arousal.

$$\text{Model 1: mean\_ISC} \sim \text{SPL\_level} + \text{SNR\_level}$$

$$\text{Model 2: mean\_ISC} \sim \text{SPL\_level} * \text{SNR\_level}$$

$$\text{Model 3: mean\_ISC} \sim \text{SPL\_level} * \text{SNR\_level} + \text{mean\_HR}$$

Model	Res.df	RSS	Df	F	Pr(>F)
1: SPL + SNR	5891	138.13	–	–	–
2: SPL × SNR	5887	137.00	4	12.20	7.03×10^{-10}***
3: SPL × SNR + HR	5886	136.71	1	12.40	0.00043***

Table S1. ANOVA table comparing nested models predicting mean heart rate ISC. Including the SPL × SNR interaction (Model 2) significantly improves model fit over additive effects (Model 1). Adding mean HR (Model 3) further improves fit but explains a smaller proportion of variance.

Model comparison was performed using ANOVA (Table S1) to assess improvements in fit between nested models. Model quality was additionally evaluated using Akaike Information Criterion (AIC) and changes in R^2. AIC values were: Model 1 = -5388.47, Model 2 = -5429.06, Model 3 = -5439.47. Marginal R^2 values were: Model 1 = 0.0185, Model 2 = 0.0265, Model 3 = 0.0286. Together, these results indicate that the interaction between SPL and SNR accounts for additional variance in heart rate ISC, whereas mean HR contributes minimally, suggesting that general arousal does not fully explain the observed effects of the sound environment.

S6. Statistical testing and model evaluation. To evaluate interpersonal physiological synchrony as indexed by inter-subject correlation of heart rate (heart rate ISC), several levels of analysis were conducted, and statistical tests were chosen based on data characteristics and experimental design. Across all analyses, normality of differences was assessed using the Shapiro–Wilk test, and appropriate parametric or non-parametric tests were selected accordingly. Effect sizes and multiple comparison corrections are reported where applicable. All statistical analyses were conducted using `R` (version 2024.04.1) and Python (version 3.8).

Group-level ISC vs. Time-misaligned shuffled controls. To test whether heart rate ISC exceeded chance levels arising from time-misaligned signals, we compared group-level heart rate ISC against time-misaligned shuffled controls. Differences were first tested for normality using the Shapiro–Wilk test. If normally distributed, paired-sample t-tests were used; otherwise, Wilcoxon signed-rank tests were applied. Effect sizes were reported as Cohen's d for t-tests and rank-biserial r for Wilcoxon tests.

Dyadic ISC across proximity conditions. At the dyadic level, heart rate ISC was computed for each pair under three physical proximity conditions: close (< 20 m), far (> 1 km), and time-misaligned shuffled controls. Normality of residuals was assessed using the Shapiro–Wilk test. Repeated-measures ANOVAs were conducted to evaluate the main effect of proximity. When the assumption of sphericity was violated, Greenhouse–Geisser correction was applied. Post hoc pairwise comparisons were conducted between all proximity conditions, with Bonferroni correction for multiple comparisons. Effect sizes are reported as partial η^2 for ANOVAs and Cohen's d for pairwise contrasts.

Social familiarity heart rate ISC. To assess the effect of social familiarity on physiological synchrony, heart rate ISC was compared between familiar (participants who knew each other prior to the trip) and unfamiliar pairs, controlling for physical proximity by including only segments where participants were in the same group. Normality of heart rate ISC differences was assessed via Shapiro–Wilk tests. When normality assumptions were met, Welch's t-tests were used; otherwise, Wilcoxon rank-sum tests were applied. Effect sizes are reported as Cohen's d for t-tests and rank-biserial r for Wilcoxon tests.

Event-level heart rate ISC analyses. To examine heart rate ISC across different social contexts, events were categorized as close-proximity interactions, stimulus-locked interactions, and dispersed interactions. For each event, heart rate ISC was averaged across all 5-minute segments for all participant pairs. Differences between observed heart rate ISC and time-misaligned controls were assessed using paired t-tests if normality was satisfied, or Wilcoxon signed-rank tests otherwise. Effect sizes were reported as Cohen's d.

S7. Controlling for mean heart rate in event-based Heart rate ISC analyses. To ensure that differences in Heart rate ISC across social contexts were not driven by overall heart rate (HR) levels, we conducted additional control analyses. For each event, individual HR time series were demeaned prior to Heart rate ISC computation, and synchrony was compared against time-misaligned shuffled controls.

We further examined the relationship between mean HR and Heart rate ISC across interaction categories to address potential arousal confounds. While the global association across all events remained non-significant ($r = 0.04, p = .45$; Fig. S1), a more detailed analysis by event type revealed:

- **Close proximity interactions**: No significant correlation was observed between mean HR and synchrony ($r = 0.08, p = .30$), despite these events being characterized by high overall arousal.
- **Dispersed interactions**: No significant relationship was found ($r = 0.15, p = .09$).
- **Stimulus-locked interactions**: While the full-group correlation was non-significant ($r = 0.16, p = .12$), a sensitivity analysis excluding two extreme Heart rate ISC outliers (> 0.25) revealed a significant positive relationship ($r = 0.36, p < .001$).

The significant correlation specifically in the stimulus-locked condition suggests that during periods of joint attention to shared stimuli (e.g., lectures or performances), higher physiological arousal may correspond to the degree of inter-subject synchronization.

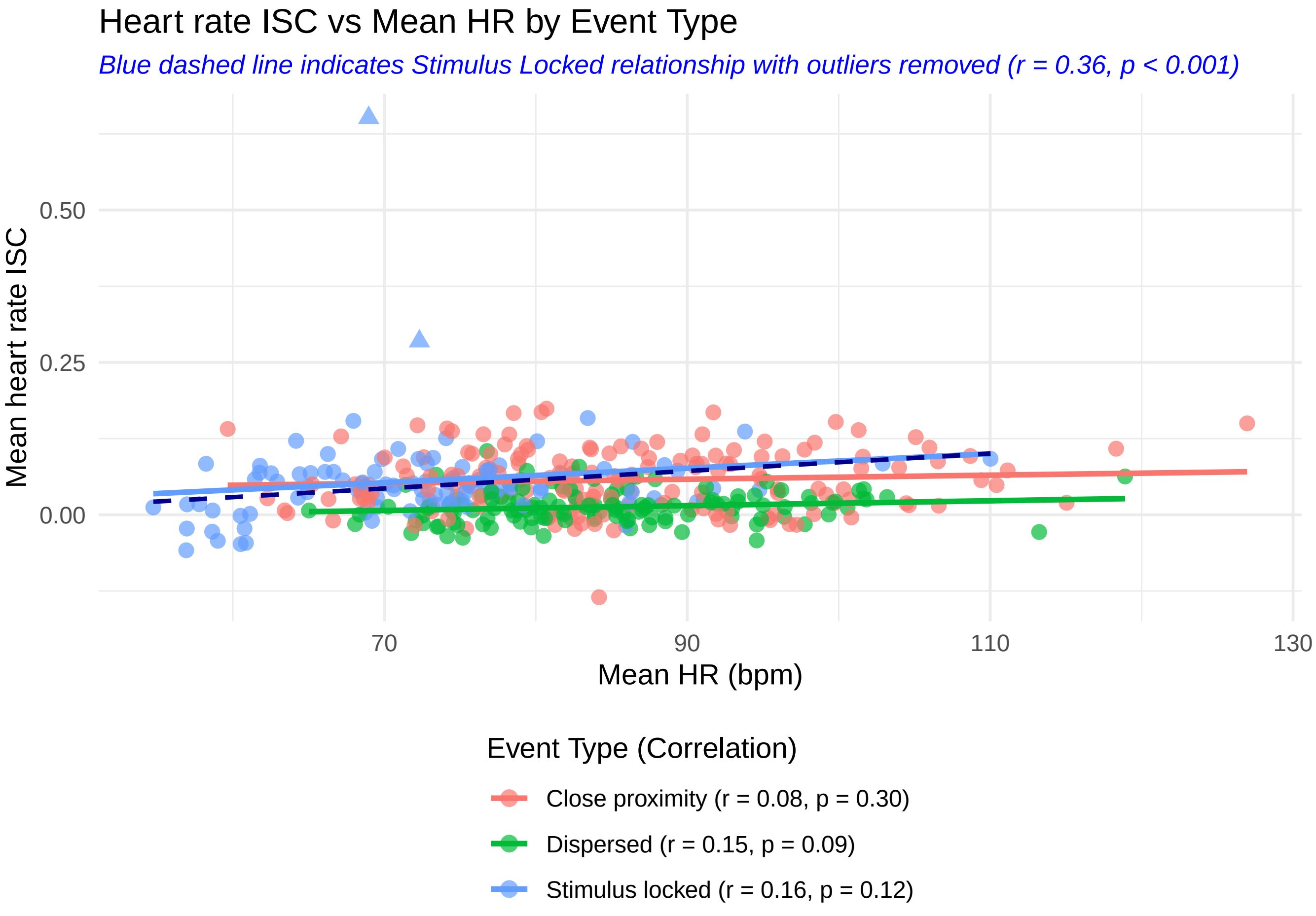

Fig. S1. Correlation between mean HR and Heart rate ISC by interaction type. The legend displays Pearson correlation coefficients (r) and p-values for each social context. The global correlation is non-significant ($r = 0.04, p = .45$). The blue dashed line represents a significant relationship within the Stimulus-locked condition when two high-ISC outliers (indicated by triangles) are excluded ($r = 0.36, p < .001$).

Finally, we assessed mean HR across event types (Fig. S2). Close proximity and dispersed interactions resulted in similarly elevated mean HR, whereas stimulus-locked events showed slightly lower mean HR. These findings show that Heart rate ISC differences across interaction types cannot be explained by global arousal alone. Close proximity and dispersed events showed similarly elevated mean HR levels, yet only the former yielded significant Heart rate ISC. Furthermore, stimulus-locked events exhibited high synchrony despite having the lowest overall mean HR across all trips.

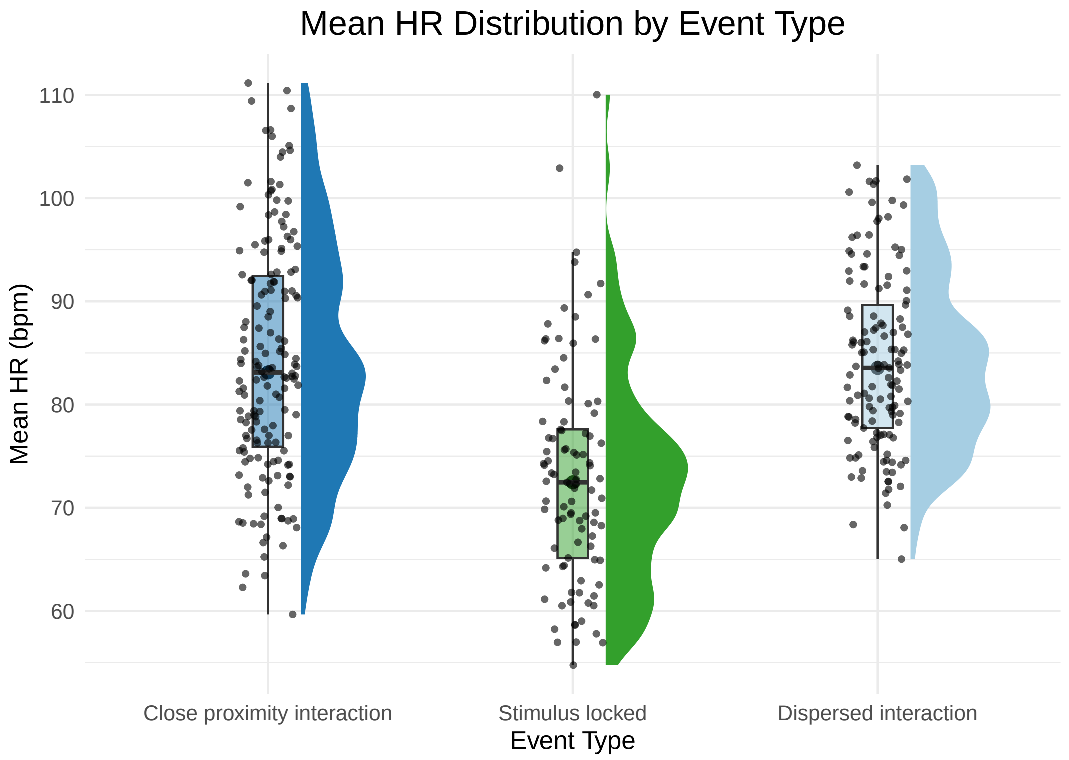

Fig. S2. Distribution of mean HR across event types. Close proximity and dispersed events showed similarly high mean HR; stimulus-locked events showed slightly lower mean HR.

S8. Heart rate ISC across all events. For completeness, we report statistical results for each event across the three trips (Tables S2–S4). Raincloud plots comparing original synchrony values with time-misaligned shuffled controls are shown in Figs. S3–S5.

Interaction Type	Event	Test	Statistic	p-value	Effect size
Close proximity	Social Dinner 1	t-test	2.17	0.047	0.54
Close proximity	Social Dinner 2	Wilcoxon	4.8	< 0.001	1.24
Stimulus-locked	Presentation 1	t-test	9.2	< 0.001	2.0
Stimulus-locked	Presentation 2	Wilcoxon	9.0	< 0.001	1.01
Dispersed	Casual Reception 1	t-test	1.09	0.31	0.36
Dispersed	Casual Dining 1	t-test	-0.35	0.73	-0.09

Table S2. Heart rate ISC statistical results for Trip 1.

Interaction Type	Event	Test	Statistic	p-value	Effect size
Close proximity	Group Game 1	Wilcoxon	22	< 0.001	1.21
Close proximity	Social Dinner 3	t-test	2.43	0.028	0.61
Stimulus-locked	Show 1	t-test	6.06	< 0.001	1.35
Stimulus-locked	Presentation 3	Wilcoxon	51	0.24	0.13
Stimulus-locked	Presentation 4	t-test	7.51	< 0.001	1.64
Dispersed	Casual Dining 2	t-test	0.683	0.5	0.17
Dispersed	Casual Reception 2	t-test	1.6	0.13	0.36

Table S3. ISC-HR statistical results for Trip 2.

Interaction Type	Event	Test	Statistic	p-value	Effect size
Close proximity	Group Game 2	t-test	5.51	< 0.001	1.26
Close proximity	Social Brunch 1	t-test	5.06	< 0.001	1.23
Stimulus-locked	Presentation 5	t-test	10.92	< 0.001	2.33
Stimulus-locked	Presentation 6	t-test	6.42	< 0.001	1.37
Stimulus-locked	Presentation 7	t-test	11.00	< 0.001	2.30
Dispersed	Casual Dining 3	t-test	2.62	0.16	0.33
Dispersed	Casual Dining 4	t-test	1.23	0.27	0.24
Dispersed	Casual Reception 3	t-test	0.98	0.03	0.52

Table S4. Heart rate ISC statistical results for Trip 3.

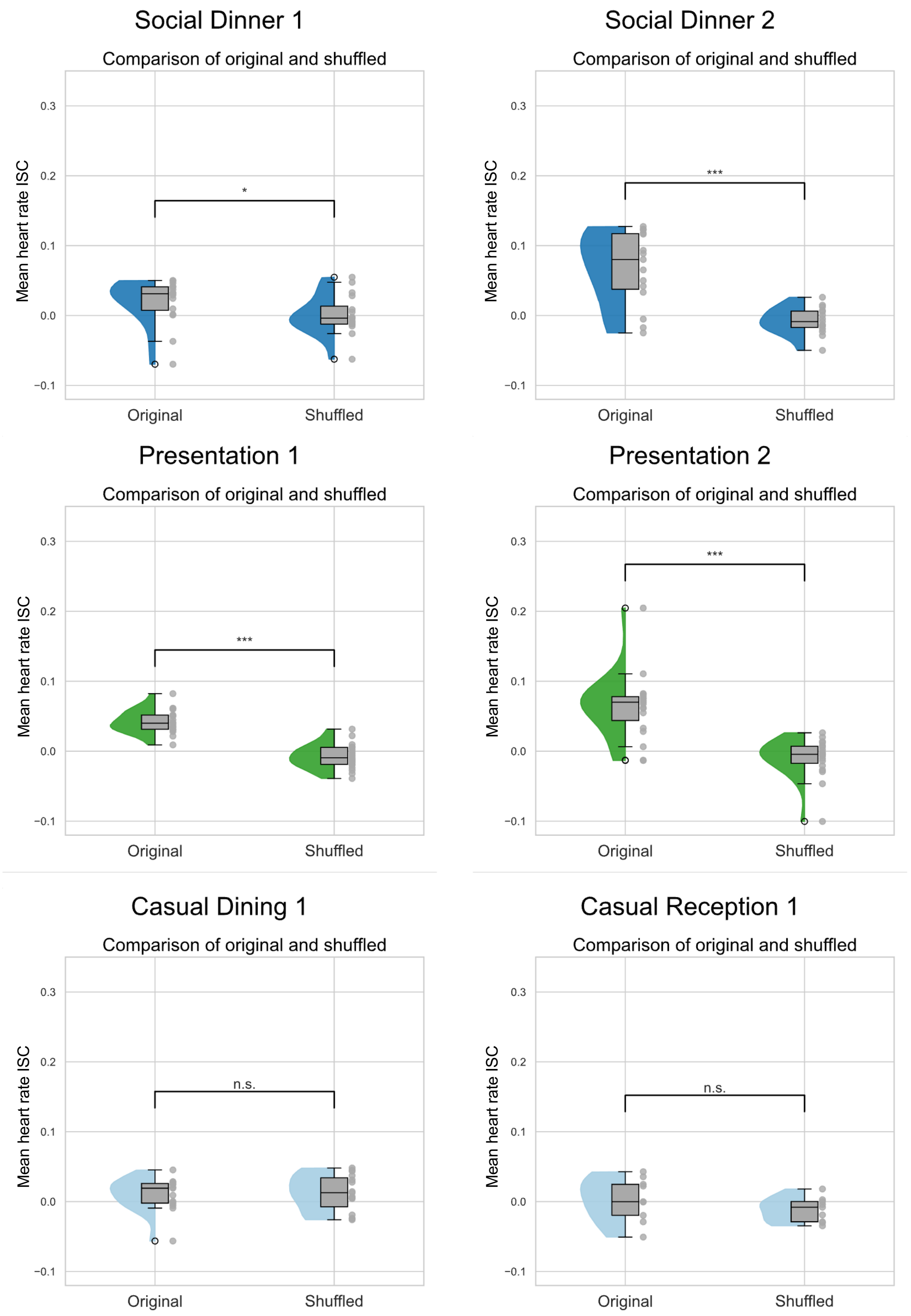

Fig. S3. Raincloud plots for Trip 1 showing original vs. time-misaligned shuffled heart rate ISC.

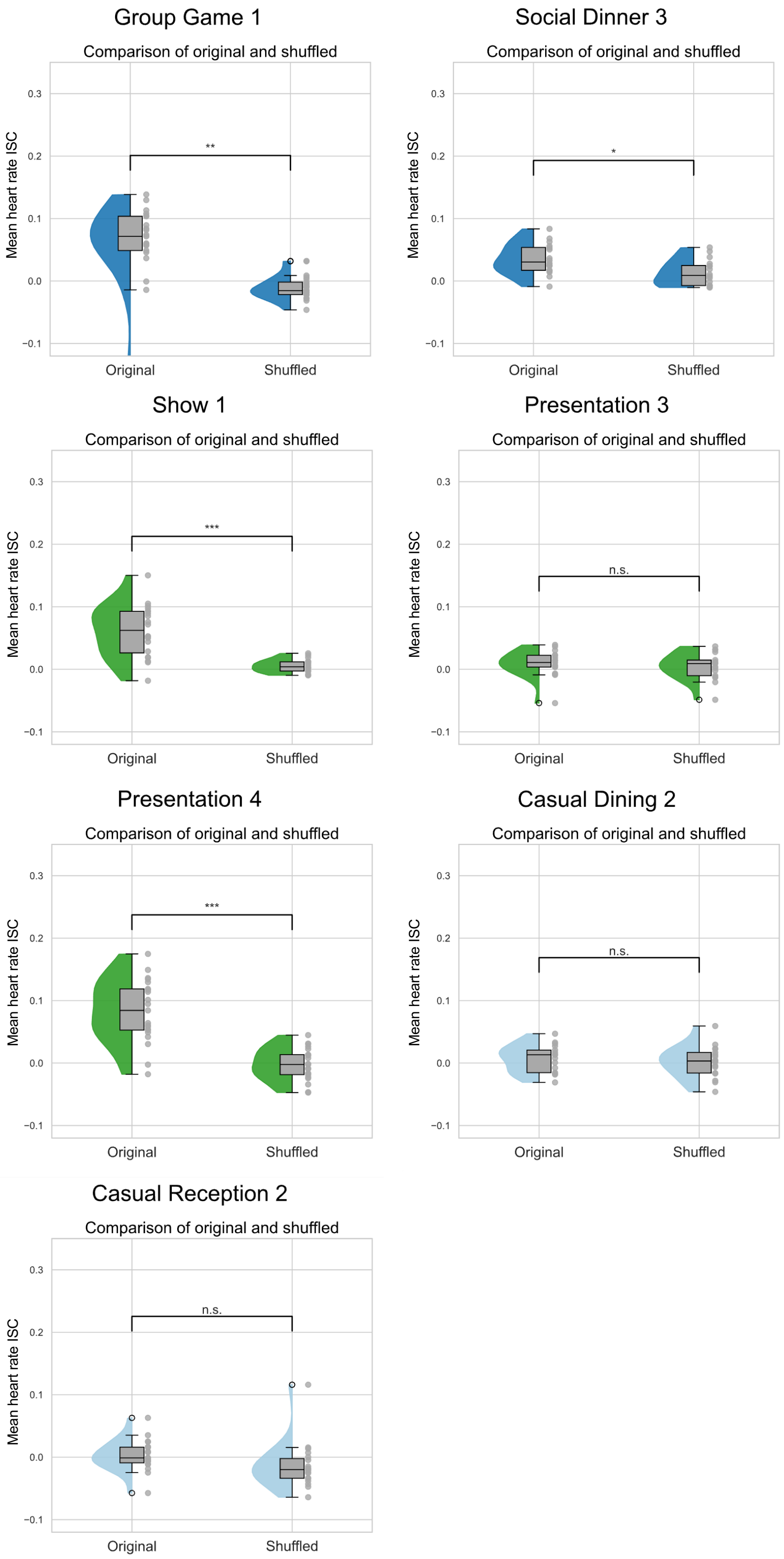

Fig. S4. Raincloud plots for Trip 2 showing original vs. time-misaligned shuffled heart rate ISC.

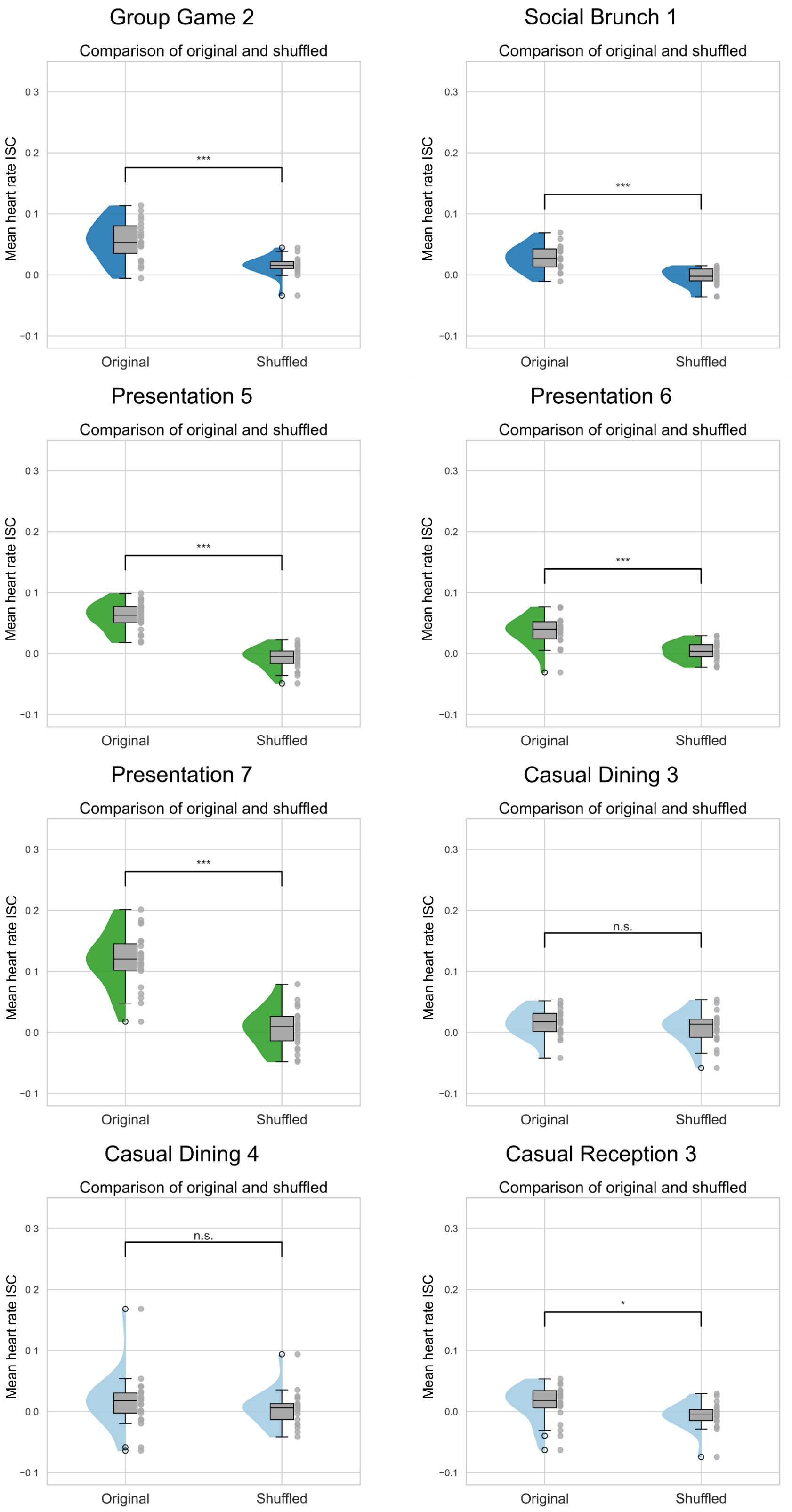

Fig. S5. Raincloud plots for Trip 3 showing original vs. time-misaligned shuffled heart rate ISC.

S9. Effect of data quality on heart rate ISC. To assess whether the observed differences in heart rate ISC across social contexts were modulated by variations in signal quality, we quantified data quality as the percentage of long gaps (intervals > 5 seconds) in the raw heart rate signal for each participant per event. This metric was calculated using the raw data prior to any pre-processing steps, such as the removal of bad segments or interpolation.

We used a linear mixed-effects model to test whether this missing data metric predicted heart rate ISC values, treating participant ID and Event as random effects to account for the nested structure of the data. The results showed no significant relationship between the percentage of long gaps and heart rate ISC ($\beta = 3.218 \times 10^{-4}$, $SE = 1.189 \times 10^{-3}$, $t(238.6) = 0.271$, $p = 0.787$).

Furthermore, a Type III ANOVA revealed that the amount of missing data did not differ significantly across the three interaction categories ($F(2, 234.11) = 1.16$, $p = 0.315$, Fig. S6). These results indicate that there is no systematic difference in heart rate synchrony explained by data quality.

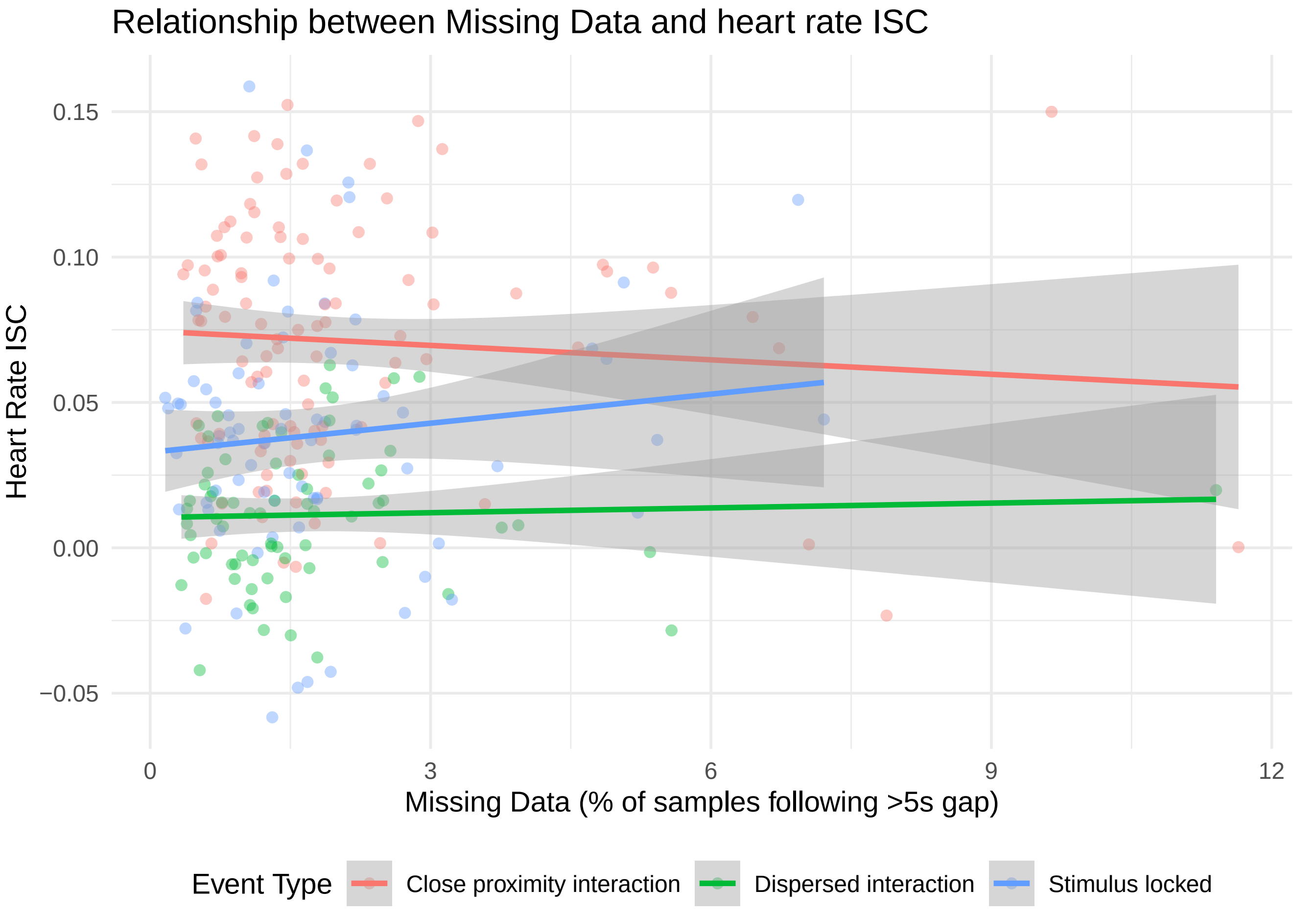

Fig. S6. Relationship between missing data and heart rate ISC. Scatter plot illustrating heart rate ISC as a function of data quality, calculated as the percentage of samples following a gap greater than 5 seconds in the raw signal per participant. Data points are color-coded by interaction category: close-proximity interaction (red), dispersed interaction (blue), and stimulus-locked (green).

References

1. J Bradley, RD Reich, S Norcross, On the combined effects of signal-to-noise ratio and room acoustics on speech intelligibility. *The J. Acoust. Soc. Am.* **106**, 1820–1828 (1999).
2. JH Christensen, GH Saunders, M Porsbo, NH Pontoppidan, The everyday acoustic environment and its association with human heart rate: evidence from real-world data logging with hearing aids and wearables. *Royal Soc. open science* **8**, 201345 (2021).
3. JM Kates, *Digital Hearing Aids.* (Plural Publishing, San Diego, CA), (2008).
4. J Crawford, L Doherty, *Practical Aspects of ECG Recording.* (M&K Update Ltd, London, UK), (2012).
5. B Bent, BA Goldstein, WA Kibbe, JP Dunn, Investigating sources of inaccuracy in wearable optical heart rate sensors. *NPJ digital medicine* **3**, 18 (2020).
6. U Hasson, Y Nir, I Levy, G Fuhrmann, R Malach, Intersubject synchronization of cortical activity during natural vision. *science* **303**, 1634–1640 (2004).
7. P Pérez, et al., Conscious processing of narrative stimuli synchronizes heart rate between individuals. *Cell Reports* **36** (2021).
8. JH Christensen, et al., Predicting individual hearing-aid preference from self-reported listening experiences in daily life. *Ear hearing* pp. 10–1097 (2024).
